# Trends, patterns and cause-specific neonatal mortality in Tanzania: a hospital-based retrospective survey

**DOI:** 10.1093/inthealth/ihaa070

**Published:** 2020-09-25

**Authors:** Chacha D Mangu, Susan F Rumisha, Emanuel P Lyimo, Irene R Mremi, Isolide S Massawe, Veneranda M Bwana, Mercy G Chiduo, Leonard E G Mboera

**Affiliations:** National Institute for Medical Research, Mbeya Research Centre, Mbeya, Tanzania; National Institute for Medical Research, Headquarters, Dar es Salaam, Tanzania; National Institute for Medical Research, Headquarters, Dar es Salaam, Tanzania; National Institute for Medical Research, Headquarters, Dar es Salaam, Tanzania; SACIDS Foundation for One Health, Sokoine University of Agriculture, Morogoro, Tanzania; National Institute for Medical Research, Tanga Research Centre, Tanga, Tanzania; National Institute for Medical Research, Amani Research Centre, Muheza, Tanzania; National Institute for Medical Research, Tanga Research Centre, Tanga, Tanzania; SACIDS Foundation for One Health, Sokoine University of Agriculture, Morogoro, Tanzania

**Keywords:** cause-specific, hospital, mortality, neonatal, Tanzania, trends

## Abstract

**Background:**

Globally, large numbers of children die shortly after birth and many of them within the first 4 wk of life. This study aimed to determine the trends, patterns and causes of neonatal mortality in hospitals in Tanzania during 2006–2015.

**Methods:**

This retrospective study involved 35 hospitals. Mortality data were extracted from inpatient registers, death registers and International Classification of Diseases-10 report forms. Annual specific hospital-based neonatal mortality rates were calculated and discussed. Two periods of 2006–2010 and 2011–2015 were assessed separately to account for data availability and interventions.

**Results:**

A total of 235 689 deaths were recorded and neonatal deaths accounted for 11.3% (n=26 630) of the deaths. The majority of neonatal deaths (87.5%) occurred in the first week of life. Overall hospital-based neonatal mortality rates increased from 2.6 in 2006 to 10.4 deaths per 1000 live births in 2015, with the early neonates contributing 90% to this rate constantly over time. The neonatal mortality rate was 3.7/1000 during 2006–2010 and 10.4/1000 during 2011–2015, both periods indicating a stagnant trend in the years between. The leading causes of early neonatal death were birth asphyxia (22.3%) and respiratory distress (20.8%), while those of late neonatal death were sepsis (29.1%) and respiratory distress (20.0%).

**Conclusion:**

The majority of neonatal deaths in Tanzania occur among the early newborns and the trend over time indicates a slow improvement. Most neonatal deaths are preventable, hence there are opportunities to reduce mortality rates with improvements in service delivery during the first 7 d and maternal care.

## Introduction

Neonatal mortality, which refers to the death of any newborn that occurs between birth and 28 d after birth, is among the major public health challenges in low- and middle-income countries.^1–3^ In 2016, an estimated 2.6 million neonatal deaths were reported representing a global burden of 19 neonatal deaths per 1000 live births, accounting for 46% of all deaths among children aged <5 y.^1^ The majority of these newborn deaths occurred in low- and middle-income countries.^1–3^ South Asia and sub-Saharan Africa (SSA) account for about three-quarters (79%) of the total burden of neonatal deaths.^3^ SSA has the highest burden of neonatal mortality with one death in every 38 newborns before the age of 1 mo.^4^ Recent statistics indicate that in 2017 the annual neonatal mortality rate (NMR) was highest in east, west and central Africa.^3^ Tanzania, Ethiopia and Nigeria are the countries with the highest NMRs in SSA.^5^ In Tanzania, population-based surveys have indicated that neonatal mortality ranged between 26 and 40 deaths per 1000 live births during the last 3 decades, with wide variations between regions.^6–9^ From 1991 to 2016, the decline in neonatal mortality was 2.2% per year.^6,9^ This reduction is likely to be due to several interventions such as immunization, introduction of integrated management of childhood illness during the first week of life, consolidation of the neonatal resuscitation program and human resource capacity enhancement in healthcare facilities.^10–13^

The determinants of neonatal mortality may be attributed to newborn, mother or health system factors. The newborn factors include age, birth weight, gender and neonatal infections.^14–16^ The maternal factors are age, parity, birth interval, education and wealth status; others consist of inadequate maternal knowledge on neonatal danger signs, complications at the time of delivery, history of abortion, a low Apgar score and home deliveries.^17,18^ Home deliveries, illiterate mothers, poor socioeconomic status, families that do not want or plan their last child and lack of continuum of care from maternal to child have been associated with neonatal mortality in a number of studies.^5,19–21^ Health system factors include antenatal care by a skilled provider, health staff attitudes, supervision of delivery and hours spent in the labor ward.^16,21–27^

Globally, one-third (35.7%) of neonatal deaths are caused by preterm birth complications followed by intrapartum complications, sepsis and other severe infections.^28,29^ The first two factors account for most early neonatal deaths while the latter causes nearly half of late neonatal deaths.^30^ The proportion of neonatal death due to each cause differs between geographical areas.^10,31,32^ In Tanzania, prematurity, asphyxia, congenital malformation and infections have been reported as the leading causes of death among neonates.^7,33^

Major efforts are being taken to address global child health challenges, particularly of neonates, to achieve national and international goals. In many SSA countries, home deliveries represent a significant proportion and most neonatal deaths occur at home and hence are neither recorded nor officially presented.^14^ In Tanzania, comprehensive neonatal mortality information depicting cause-specific patterns and trends is lacking. The dearth of such important information limits the use of mortality data for developing appropriate interventions and informing evidence-based policy decisions. Hospital statistics present a great opportunity to study the trends, patterns and causes of neonatal mortality. Hospital mortality data provide information on disease burden and are a way of exploring the performance of the health system.^34^ Despite their limitation in estimating generalized mortality statistics, hospital data are reliable as causes of death are confirmed by qualified health personnel based on direct events that occurred to the patient, hence are not disadvantaged by recall bias as those reported via verbal autopsy.^35^ NMR is considered to be one of the key indicators of the health status of a community as it reflects the quality of prenatal, delivery and early infant care available in the country.^31^ Therefore, this study was conducted to determine the burden, patterns, trends and causes of neonatal mortality in selected hospitals of Tanzania during 2006–2015.

## Methods

### Study design and setting

This study was carried out from July to December 2016 involving district, regional referral, zonal referral and national hospitals. To sample the hospitals, the administrative regions were categorized into three strata based on three criteria. First, the proportional contribution to the national population of either a high populated region (Dar es Salaam, Mwanza, Mbeya), medium populated region (Kagera, Tabora, Morogoro, Kigoma, Dodoma, Tanga) or low populated region (Arusha, Geita, Iringa, Katavi, Kilimanjaro, Lindi, Manyara, Mara, Mtwara, Njombe, Pwani, Rukwa, Ruvuma, Shinyanga, Singida, Simiyu) was calculated.^35^ Second, the distribution of the hospitals within the country and regions was assessed to ensure representation (http://hfrportal.moh.go.tz/). Third, the epidemiological burden and spatial variations of malaria and HIV/AIDS endemicity, child mortality and human resource coverage were assessed.^9^ The stratification resulted in a decision to include three hospitals in areas with high, two hospitals for medium and one hospital for low population density. The study sites were selected to ensure geographical representation for all regions in the country.

Thirty-five hospitals were selected for the study. These consisted of 1 national hospital (Muhimbili National Hospital), 3 zonal referral hospitals (Mbeya, Bugando Medical Centre and Kilimanjaro Christian Medical Centre), 20 regional referral hospitals (to create regional representation in exclusion of regions where the national or zonal referral hospital was located; Temeke, Kagera, Kitete–Tabora, Morogoro, Maweni–Kigoma, Dodoma, Bombo-Tanga, Mara, Mount Meru–Arusha, Shinyanga, Manyara, Ruvuma, Singida, Geita, Ligula–Mtwara, Tumbi–Pwani, Rukwa, Iringa, Sokoine–Lindi and Njombe) and 11 district hospitals (Sengerema, Ukerewe, Mpanda, Kyela, Chunya, Biharamulo, Nzega, Kilosa, Kibondo, Lushoto and Maswa), to complete the necessary number of hospitals for high and medium populated regions. The district hospitals were randomly selected within a region, excluding those districts where the regional or zonal hospital was located. The selected hospitals constituted about 15% of all the hospitals in the country at the time of the survey.

### Data source

Mortality data were extracted from inpatient department registers, death registers and International Classification of Diseases (ICD)-10 report forms. A thorough search and compilation of all identified registers and forms used to record mortality data was conducted. The hospital records were extracted manually from sources and data were compiled using a customized paper-based data collection tool. This study utilized all death records of individuals aged 0–28 d. The variables collected were the deceased's age, gender, cause and date of death. Three research physicians trained on the cause of death classification worked independently to group and code the cause of death according to ICD-10. Where there was a discrepancy, a consensus was sought among them and the cause of death was coded accordingly. Details of data extraction have been described elsewhere.^35,36^

### Data analysis

Data were entered and processed in Epi-Data software (EpiData Association, 2010; Odense, Denmark). Data were analyzed using STATA version 14 (StataCorp LP, College Station, TX, USA). Variables of interest were causes of death and age at death classified as early neonatal (0–6 d) and late neonatal (7–28 d) periods. Causes of Death and Associated Conditions (CODAC) rules for classifying perinatal and neonatal deaths^38^ were used to present the single and associated cause of death where multiple causes were recorded.

Patterns of neonatal causes of death were studied by year, region and level of hospital. To interpret the time trend of neonatal deaths, the 10-y study duration was divided into 2006–2010 and 2011–2015 periods. This was done for the following reasons. First, to track the country's achievements and progress towards the Sustainable Development Goal (SDG) on neonatal mortality, that is, to end preventable deaths of newborns, with countries aiming to reduce neonatal mortality to at least 12 deaths per 1000 live births by 2030. The United Nations 2011 estimate for Tanzania stands at 25/1000 live births (http://data.un.org), which means the target is to halve the rate. Second, since 2006 there have been several initiatives to improve the quality of maternal and newborn care in the country,^12,39–43^ however, the largest part of reporting was paper-based with few attempts to monitor data quality, thus affecting the completeness of data gathered during 2006–2010.^37^

We calculated annual hospital-based NMRs expressed as deaths per 1000 live births to assess the trend from 2006 to 2015. Using the statistics on institutional deliveries from the nationally representative demographic and health surveys^6,9^ and the estimated proportion of births that occurred in hospitals,^41^ we deducted from statistics taken from World Development Indicators databases (data.worldbank.org) to obtain the close-to-correct hospital-based live births denominator for our calculations. Population statistics are used here because we could not obtain complete actual data on live births for all hospitals in Tanzania. It is estimated that at least half of all births occurred in health facilities and among those 40% occurred in hospitals. Early NMR and late NMR were calculated and compared separately.

## Results

### Trend and NMR

**Figure 1.**
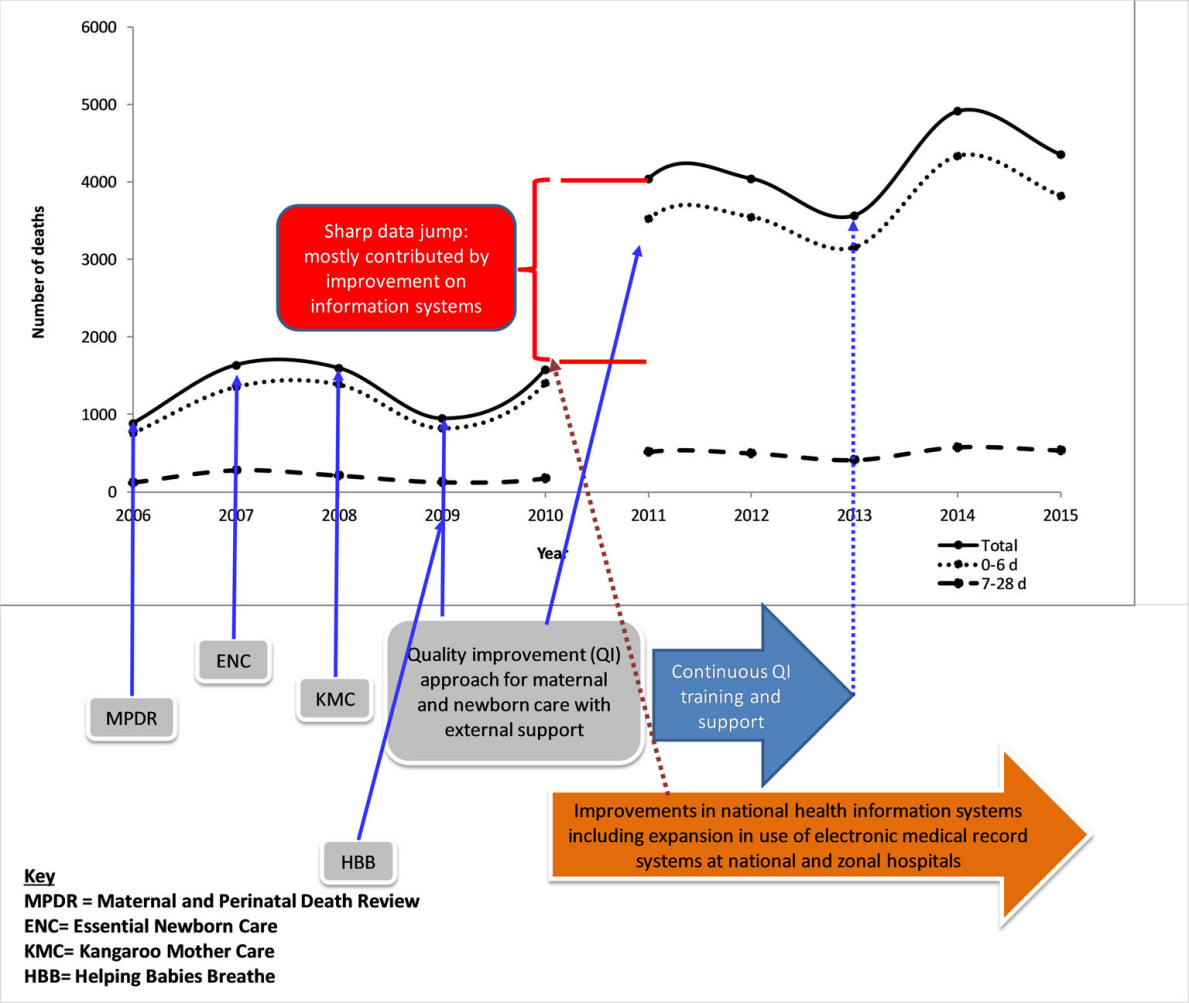
Number of total, early (0–6 d) and late (7–28 d) hospital-based neonatal deaths, 2006–2015.

A total of 235 689 deaths were recorded in 35 hospitals during the 10-y period. Of the total deaths, 26 630 (11.3%) were among neonates. Male neonates accounted for the majority of deaths (54.7%; n=14 566). Among the neonatal deaths, 23 590 (88.6%) occurred during early neonatal life. There was a marked annual increase in neonatal deaths from 883 in 2006 to 4353 in 2015 with a substantial rise after the first 5 y (Figure 1). The average number of annual neonatal deaths from 2006 to 2010 was 1329 (lowest=883, highest=1597) while during 2011–2015 it was 4182 (lowest=3567, highest=4910).

The mortality trends differed between the early and late neonatal periods. Early neonatal deaths fluctuated with clear variation between years while late neonatal deaths displayed a steady pattern. Overall, there were two interesting trends observed. First, there was a decline in the number of deaths by 40.7% from 1597 in 2006 to 947 in 2009 followed by a sharp increase of 66.8% (n=1580) in 2010. Second, there was an increase of 37.6% from 2013 to 2014 followed by a decline of 11.3% from 2014 to 2015.

**Figure 2.**
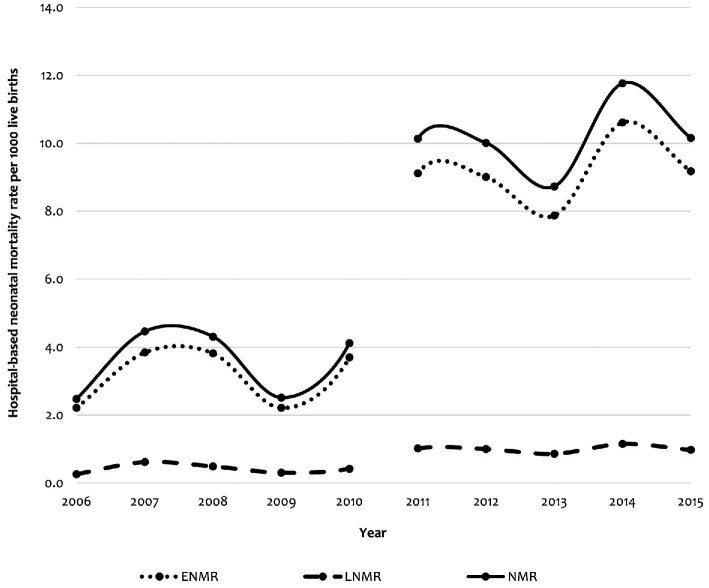
Hospital-based neonatal mortality rates for all neonates, early (0–6 d) and late (7–28 d), 2006–2015.

The overall hospital-based NMR for 2006–2015 was estimated to be 7.2 per 1000 live births. The rate was 3.7 per 1000 live births for 2006–2010 and 10.4 per 1000 live births for 2011–2015. The overall NMR increased from 2.6 in 2006 to 3.2 per 1000 live births in 2010, then the rate rose sharply to 10.4 per 1000 live births in 2011, which remained almost stable until 2015, with the exception of an increase from 2013 to 2014. This pattern was similar for both early and total neonatal deaths. Overall, the late NMR remained stable over the years (Figure 2).

### Causes of neonatal mortality

The leading causes of death in the early neonatal period were birth asphyxia (22.3%, n=5255), neonatal respiratory distress (20.8%, n=4896), prematurity (12.0%, n=2833) and neonatal sepsis (11.6%, n=2745). Other neonatal infections, which included malaria, pneumonia and meningitis, accounted for 7.4% (n=1743) of all early neonatal deaths. Maternal complications contributed 8.6% (n=2043) of all early neonatal deaths. On the other hand, the leading causes of death among the late neonates (7–28 d) were sepsis (29.1%, n=885), respiratory distress (20.0%, n=609), other infections (19.8%, n=603) and prematurity (10.6%, n=322). Sepsis and other neonatal infections accounted for 48.9% of all late neonatal deaths (Table 1). The cause of death was not specified in 7.8% (n=1840) of reported deaths, with the majority (78.0%) reported as cardiorespiratory failure.

**Table 1.** Specific causes of death among early and late neonates, 2006–2015

	Frequency (%)		
Variable	Early neonates (n=23 590)	Late neonates (n=3040)	Total neonates (N=26 630)
Neonatal causes			
Asphyxia	5255 (22.3)	178 (5.9)	5433 (20.4)
Neonatal respiratory distress	4896 (20.8)	609 (20.0)	5505 (20.7)
Prematurity	2833 (12.0)	322 (10.6)	2785 (10.5)
Neonatal sepsis	2745 (11.6)	885 (29.1)	3630 (13.6)
Neonatal infections	1743 (7.4)	603 (19.8)	2346 (8.8)
Encephalopathy	899 (3.8)	26 (0.9)	925 (3.5)
Hemolytic disorders of the newborn	408 (1.7)	102 (3.4)	510 (1.9)
Congenital malformation	426 (1.8)	55 (1.8)	481 (1.8)
Low birthweight	187 (0.8)	34 (1.1)	591 (2.2)
Metabolic conditions	162 (0.7)	17 (0.6)	179 (0.7)
Organ failure	36 (0.2)	19 (0.6)	55 (0.2)
Hypothermia	44 (0.2)	0	44 (0.2)
Birth injuries	55 (0.2)	1 (0.03)	56 (0.2)
Fetal intoxication	3 (0.01)	0	3 (0.01)
Necrotizing enterocolitis	15 (0.1)	0	15 (0.1)
Unspecified	1840 (7.8)	172 (5.9)	2012 (7.6)
Maternal causes			
Maternal placenta, cord and memberane	1043 (4.4)	5 (0.2)	1048 (3.9)
Maternal medical conditions	639 (2.7)	9 (0.3)	648 (2.4)
Maternal labor and delivery	352 (1.5)	3 (0.1)	355 (91.3)
Maternal obstetric causes	9 (0.04)	0	9 (0.03)

**Figure 3.**
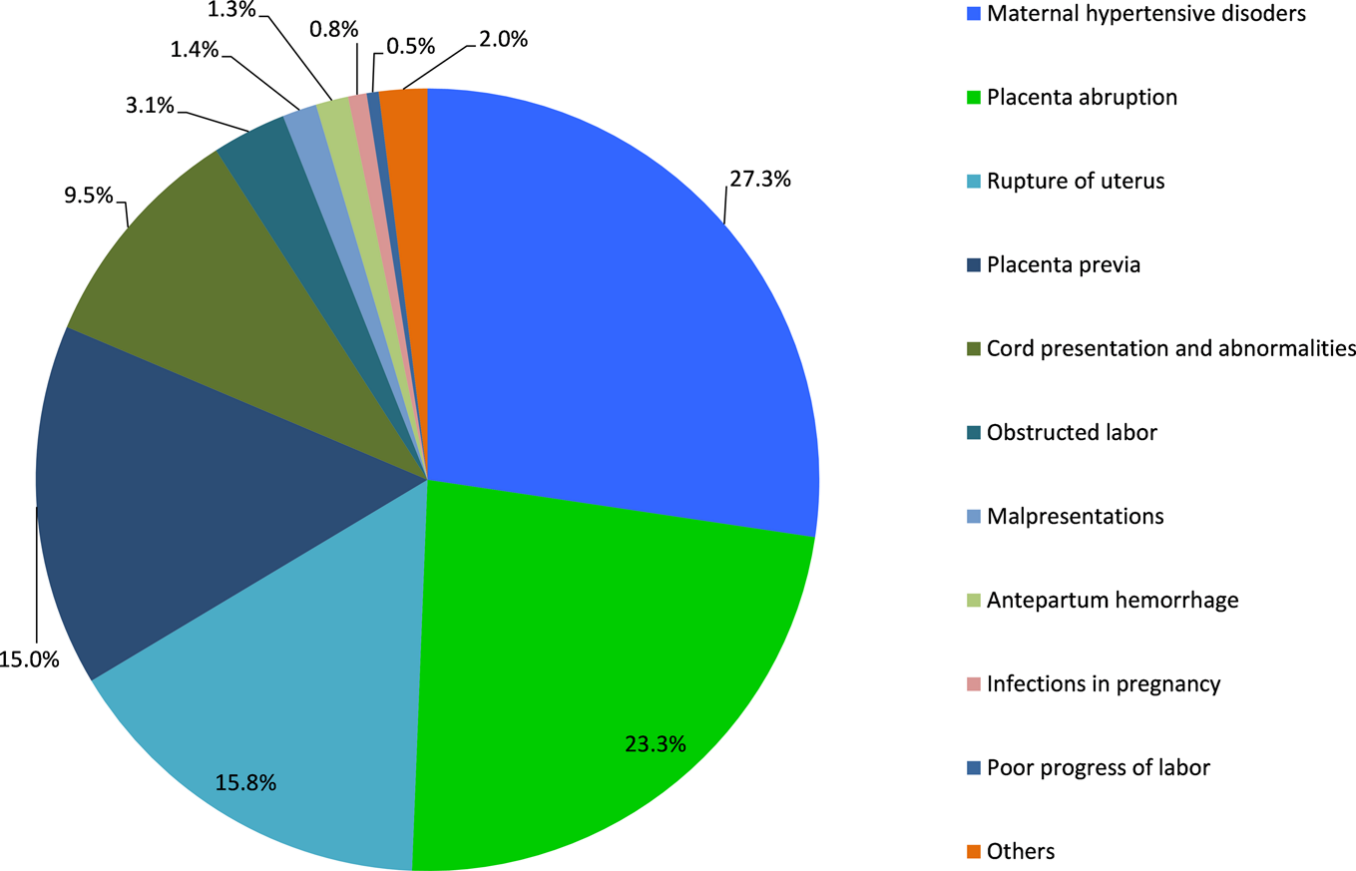
Specific maternal conditions associated with early neonatal death, 2006–2015.

Among early neonates, malaria accounted for 70% of all specific infections. The others were pneumonia (27%), meningitis (2%) and undetermined causes (1%). Similarly, among late neonates, malaria accounted for 69% of all infections followed by pneumonia (26%), meningitis (4%) and other infections (1%). One case of congenital rubella syndrome and three cases of neonatal tetanus were recorded. The most frequent congenital malformation conditions among early neonates were omphalocele and gastroschisis while among late neonates these were omphalocele and spina bifida. The most frequent specific maternal conditions associated with neonatal deaths were hypertensive disorders, placenta abruption, rupture of the uterus and placenta previa (Figure 3).

About a quarter (25.1%) of all primary causes of neonatal death were recorded together with associated causes. Using CODAC rules for classifying perinatal and neonatal deaths,^33^ birth asphyxia was the most frequent associated cause of death among the top five causes, which also accounted for >80% of all causes of deaths (Table 2). Others were prematurity and maternal complications. While maternal complication was frequently reported as an associated cause of death in early neonatal deaths, congenital abnormalities and bleeding disorders, although relatively rare, were frequently reported in late neonatal deaths. Birth injury was the least associated cause of death among the reported primary causes of neonatal death.

**Table 2.** Associated causes of deaths (columns) for each primary cause of death (first row) for early and late neonates

Birth asphyxia		Respiratory distress		Neonatal sepsis		Prematurity		Other neonatal infections		Encephalopathy	
Early neonates											
Maternal complication	58.4%	Prematurity	41.1%	Birth asphyxia	27.6%	Birth asphyxia	69.6%	Organ failure	35.2%	Birth asphyxia	47.8%
Congenital abnormalities	14.5%	Birth asphyxia	14.0%	Prematurity	25.1%	Hypothermia	7.7%	Birth asphyxia	21.3%	Prematurity	22.7%
Bleeding disorders	10.4%	Encephalopathy	8.7%	Bleeding disorders	18.9%	Maternal complications	6.9%	Neonatal sepsis	21.1%	Neonatal infection	12.5%
Metabolic disorders	7.4%	Maternal complication	7.7%	Maternal complications	12.3%	Bleeding disorders	4.5%	Prematurity	12.1%	Neonatal sepsis	9.1%
Birth Injury	6.3%	Neonatal infection	7.6%	Metabolic disorders	7.7%	Metabolic disorders	4.0%	Congenital abnormalities	3.3%	Maternal complications	2.6%
Other	3.0%	Other	20.9%	Other	8.4%	Other	7.3%	0ther	7.0%	Other	5.3%
Late neonates											
Metabolic disorders	74.1%	Prematurity	47.6%	Bleeding disorders	32.9%	Birth asphyxia	75.5%	Neonatal sepsis	58.9%	Birth asphyxia	68.2%
Bleeding disorders	14.8%	Neonatal infection	15.2%	Prematurity	27.2%	Bleeding disorders	18.4%	Prematurity	13.5%	Prematurity	9.1%
Congenital abnormalities	3.7%	Neonatal sepsis	13.3%	Birth asphyxia	13.9%	Metabolic disorders	4.1%	Bleeding disorders	10.9%	Bleeding disorders	9.1%
Birth injury	3.7%	Birth asphyxia	7.1%	Metabolic disorders	10.1%	Congenital abnormalities	2.0%	Birth asphyxia	9.6%	Low birthweight	9.1%
Organ failure	3.7%			Congenital abnormalities	6.3%			Congenital abnormalities	5.1%	Neonatal sepsis	4.5%
Other	-	Other	16.8%	Other	9.6%	Other	-	Other	2.0%	Other	-

### Mortality pattern by hospital level and geographical area

**Figure 4.**
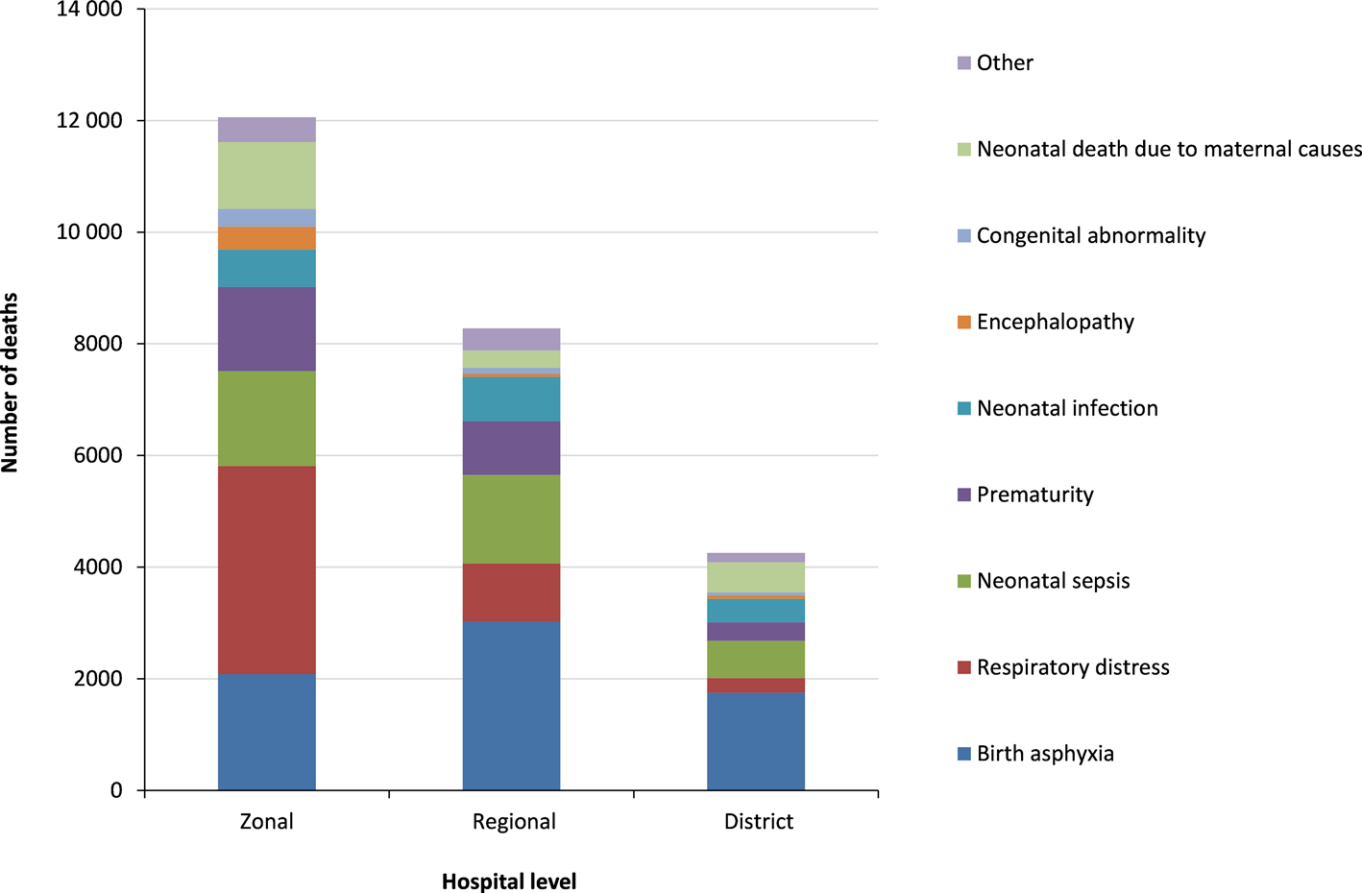
Causes of neonatal death (early and late) by hospital level, 2006–2015.

**Figure 5.**
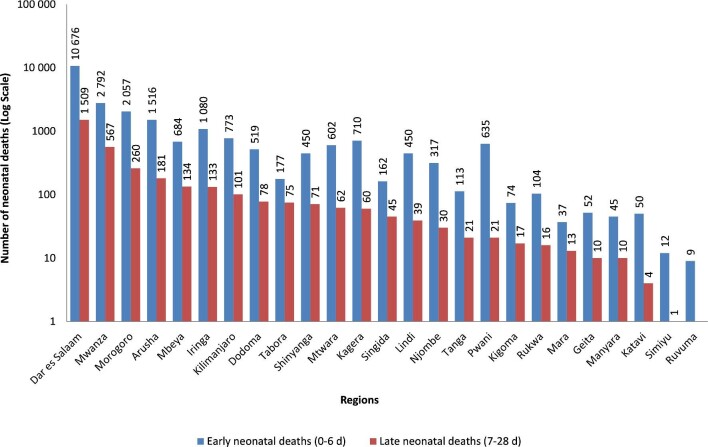
The number of hospital early and late neonatal deaths by geographical regions, 2006–2015.

Causes of neonatal death varied between hospital levels. Forty percent of the recorded deaths occurred in the zonal referral hospitals, with respiratory distress being the leading cause of death followed by birth asphyxia, neonatal sepsis and prematurity. Birth asphyxia caused more death in regional referral and district hospitals (Figure 4). There were considerable variations in the number of neonatal deaths among geographical regions. Hospitals in Dar es Salaam, Mwanza, Morogoro and Arusha accounted for more than two-thirds (60.7%) (n=18 558) of all neonatal deaths reported. The Simiyu and Ruvuma regions reported the lowest number of neonatal deaths, mainly affecting early neonates (Figure 5).

## Discussion

Neonatal death accounted for 11.3% of all in-hospital deaths in Tanzania. The majority of deaths (87.5%) occurred among early neonates. There was a substantial increase in neonatal mortality from 2006 to 2015. The leading causes of early neonatal death were birth asphyxia, respiratory distress, prematurity and neonatal sepsis. Sepsis, respiratory distress and malaria were the leading causes of late neonatal mortality.

Overall, the in-hospital neonatal mortality recorded in Tanzania demonstrated an apparent stagnation during the 10-y period, with similar patterns observed in 2006–2010 and 2011–2015. However, for both periods, hospital-based neonatal mortality fluctuated between years but maintained the same range of between approximately 900 and 1500 deaths for 2006–2010 and approximately 3500 and 5000 deaths for 2011–2015, with no sign of significant reduction. A similar pattern was observed for estimated hospital-based NMRs. The findings in this study indicate clearly that the number of in-hospital neonatal deaths continues unabated despite the implementation of various interventions.^36–39^ During almost the same period of time (2005–2015), population-based surveys have indicated that the NMR in Tanzania remained relatively unchanged,^42^ declining at a slightly slower rate of 2.2% per year.^6,9^ Other studies have reported that Tanzania has registered substantial progress in reducing child mortality but not in neonatal mortality.^8,11,35^ A recent systematic review reported that globally, from 1990 to 2017, the NMR decreased by 51%, although it varied widely.^3^ These findings indicate that Tanzania will achieve the SDG target at a slow rate. Although the reasons for the stagnation in hospital neonatal mortality in the current study could not be established, some sociocultural and health system factors are likely to play a role in Tanzania^10,11^ and other low-income countries.^15,21,44–46^ Several gaps that negatively affect neonatal health interventions in Tanzania have been identified, including the availability of supplies, equipment, human resources, funding and medicines as well as poor effective coverage of services targeting neonatal survival.^42^ It is important to note that efforts to reduce neonatal mortality should depend on efforts to strengthen health systems in the country.^47^

Our study has demonstrated that >75% of neonatal deaths occurred during the first 7 d of life. Few studies have reported the timing of neonatal deaths in Tanzania.^7,47^ A population-based study in southeastern Tanzania reported that 61% of the neonatal deaths occurred during the first 3 d of birth.^45^ A previous review indicated that about 50% of newborn deaths in Tanzania occur in the first 24 h of life while >75% of them occur during the first week of life.^48^ The findings of the current study correlate with reports from studies elsewhere in SSA.^16,49–52^ Available statistics indicate that about two-thirds (62%) of all neonatal deaths occur during the first 3 d of life.^53^ Information on the timing of neonatal death is crucial as it may provide policy and decision-makers with evidence for designing optimal delivery strategies of effective interventions to address neonatal mortalities.

In this study, birth asphyxia, respiratory distress, prematurity and sepsis were the leading causes of early neonatal mortality, contributing >75% of all causes. On the other hand, infections (including sepsis) and respiratory distress were the most commonly associated causes of death among late neonates. Prematurity, asphyxia and infections have been reported as the three leading causes of neonatal death in community-based surveys in Tanzania.^9,33,45,54^ Prematurity is the leading cause of neonatal mortality worldwide, causing >1 million deaths annually^55^ and accounting for >25% of neonatal deaths.^14^ Prematurity and birth asphyxia are closely linked to maternal health. It is estimated that >60% of all preterm births that occur in SSA are associated with high fertility and consequently a large number of births.^55^ Similar to our findings, birth asphyxia, prematurity and neonatal sepsis account for the majority of early neonatal deaths while infections cause most of the late neonatal deaths in other regions of the world.^1,16,30,56–58^ Sepsis, malaria and pneumonia were among the most important infectious diseases of the neonates in this study. Sepsis has been reported as the most common cause of death among neonates in other studies in Tanzania.^7,52^ Some studies have reported that congenital and/or acquired neonatal malaria are prevalent in Tanzania.^59–61^

Contrary to our expectations, we demonstrated a larger proportion of death occuring in the higher level of the referral system, where the quality of care and expertise are expected to be high. The reason for this could be delay or inappropriate obstetric referrals from the lower level hospitals, which suggests late arrivals for life-saving interventions or overwhelmed referral facilities that compromise the quality of care.^62–64^ Also, it may be that most of the neonates referred to these hospitals are in a critical condition, thus, in the absence of sufficient comprehensive obstetrics care, are subject to a low survival rate. A recent facility-based study in Tanzania reported that provision of emergency obstetrics care is inadequate and is mainly compromised by policy restrictions, lack of supplies and professional development, as well as by operating under poorly developed referral services.^65^ Moreover, it has been recently reported that the distribution of emergency obstetrics and neonatal care facilities in Tanzania is suboptimal in more than half of the regions with clustering around cities and townships.^63^ Higher neonatal mortalities are common in countries with low skilled attendance and institutional delivery rates.^17^ The regional variation in the cause of death could be attributed to either proximal, neonatal or health system-related factors or a combination of these.^17–19^ A recent study in Tanzania has shown that there are large regional differences in health intervention coverage and these variations are related to differences in socioeconomic development and the strength of health systems among the regions of Tanzania.^64^

Our study has some limitations, one of which is poor adherence to and non-adherence to ICD nomenclature, leading to poorly documented causes of death, as described elsewhere.^36,37^ Since a proportion of deliveries in Tanzania occur at home and those causes of death are not documented, hospital data are likely to provide an incomplete picture of the burden of neonatal mortality. Despite these limitations, this study consolidates information on neonatal mortality and highlights its trends and patterns in Tanzania.

### Conclusions

Overall, the neonatal mortality trend in Tanzania demonstrates an apparent stagnation during the 10-y period, with similar patterns observed in 2006–2010 and 2011–2015 despite interventions instituted during the early 2010s. Tanzania has a strong commitment to reducing neonatal mortality towards attaining the SDG, which is likely to have resulted in stability regarding the trend of hospital neonatal deaths from 2011 to 2015. The first 7 d of life contribute to the majority of all in-hospital neonatal deaths in Tanzania. While the leading causes of early neonatal mortality were birth asphyxia, respiratory distress, prematurity and sepsis, the leading cause of late neonatal mortality was infectious diseases, including malaria and pneumonia. The most common causes of neonatal mortality are either preventable by improving obstetric and delivery care or managed with simple and affordable interventions. It is therefore important to emphasize maintaining the improvements made in prenatal and intrapartum care, obstetric emergency services and postnatal care to ensure that neonatal mortality in the early days of life is reduced in Tanzania. Monitoring trends and cause-specific neonatal mortality in hospitals is likely to inform the quality of care and hence guide management decisions. The results of this study provide important evidence for health policy development and interventions. These findings can be used by governments and hospitals to identify major neonatal health problems and to facilitate priority-setting.

## Data Availability

None
